# Effect of Information and Communication Technology–Based Self-management System DialBeticsLite on Treating Abdominal Obesity in the Specific Health Guidance in Japan: Randomized Controlled Trial

**DOI:** 10.2196/33852

**Published:** 2022-03-24

**Authors:** Masahiro Kondo, Teru Okitsu, Kayo Waki, Toshimasa Yamauchi, Masaomi Nangaku, Kazuhiko Ohe

**Affiliations:** 1 Department of Planning, Information and Management University of Tokyo Hospital Tokyo Japan; 2 Graduate School of Interdisciplinary Information Studies The University of Tokyo Tokyo Japan; 3 Institute of Industrial Science The University of Tokyo Tokyo Japan; 4 Department of Biomedical Informatics Graduate School of Medicine The University of Tokyo Tokyo Japan; 5 Department of Diabetes and Metabolic Diseases Graduate School of Medicine The University of Tokyo Tokyo Japan; 6 Division of Nephrology and Endocrinology Graduate School of Medicine The University of Tokyo Tokyo Japan

**Keywords:** mHealth, smartphone app, abdominal obesity, self-management, telemedicine, digital health, app, obesity, overweight, weight, randomized controlled trial, intervention, lifestyle, behavior, mobile phone

## Abstract

**Background:**

Mobile health (mHealth) interventions, a more cost-effective approach compared with traditional methods of delivering lifestyle coaching in person, have been shown to improve physical parameters and lifestyle behavior among overweight populations. In Japan, the Specific Health Checkups and Specific Health Guidance (SHG) started in 2008 to treat obesity and abdominal obesity. However, the effectiveness of SHG is limited owing to its in-person counseling. The effect of mHealth on SHG has yet to be demonstrated.

**Objective:**

This study aims to determine whether a mobile self-management app (DialBeticsLite) could make the SHG more beneficial among patients with abdominal obesity to achieve a reduction in visceral fat area (VFA).

**Methods:**

This study was an open-label, 2-arm, parallel-design randomized controlled trial. We recruited 122 people in September 2017 and randomly assigned them into either the intervention or control group. All participants attended an educational group session that delivered information regarding diet and exercise. In addition, participants in the intervention group were asked to use DialBeticsLite for 3 months. DialBeticsLite facilitated the daily recording of several physical parameters and lifestyle behavior and provided feedback to encourage an improvement in behavior. The primary outcome was the change in VFA from baseline to the 3-month follow-up. Secondary outcomes included changes in both physical and metabolic parameters from baseline to the 3-month follow-up. The Welch 2-tailed *t* test was conducted to analyze the effects of DialBeticsLite on both the primary and secondary outcomes.

**Results:**

Of the 122 participants recruited, 75 (61.5%) were analyzed because 47 (38.5%) were excluded: 37 (30.3%) because of ineligibility and 10 (8.2%) because of withdrawal of consent. The mean age was 49.3 (SD 6.1) years in the intervention group (41/75, 55%) and 48.5 (SD 5.3) years in the control group (34/75, 45%), and all participants were men, although unintentionally. The baseline characteristics did not differ significantly between the intervention and control groups, except for VFA. The average change of VFA was −23.5 (SD 20.6) cm^2^ in the intervention group and +1.9 (SD 16.2) cm^2^ in the control group (*P*<.001). Statistically significant differences were also found for the change of body weight, BMI, and waist circumference. These findings did not change after adjusting for VFA at the baseline. The intervention had no significant effect on any of the metabolic parameters. An exploratory analysis showed significant associations between the change in VFA and steps per day and between the change in VFA and calorie intake per day within the intervention group.

**Conclusions:**

Our findings indicate that an mHealth intervention facilitating the daily monitoring of several physical parameters and lifestyle behavior can be highly effective in inducing visceral fat loss and weight loss among adults eligible for SHG.

**Trial Registration:**

UMIN Clinical Trials Registry UMIN000042045; https://tinyurl.com/4vat3v53

## Introduction

### Background

Obesity is an increasingly concerning epidemic that is in need of urgent management [1], as its prevalence and the comorbidities and mortality associated with it have been, and continue to, rise rapidly worldwide [2-6]. It has also tremendously increased the global health burden and health care cost [1,5,7,8]. Abdominal obesity (AO), which is defined by the amount of visceral fat, is especially known as a risk factor for cardiovascular disease, diabetes, and fatty liver disease [9-12]. Lifestyle and behavioral interventions are considered effective for improving AO [13,14]. In recent years, nationwide lifestyle interventions have been implemented in several countries [15,16]. In Japan, the Specific Health Checkups and Specific Health Guidance started in 2008. All health insurers in Japan were required to offer health screening programs to all enrollees and their dependents aged between 40 and 74 years and to provide lifestyle counseling to participants who were not taking medication but who had AO or obesity and were at risk for metabolic syndrome. These programs have been shown to successfully lead to weight loss and improvements in several other physical parameters [16]. However, behavior changes are usually difficult to achieve in wide-scale clinical practice because of limited resources and professional support [17,18]. The Specific Health Guidance suffers from suboptimal participation, with only 23.2% of those asked to enroll in the guidance completing the course in 2019 [19]. There is the problem that there is great disparity of implementation rates between different insurers, as well as a likely variation of intervention intensity due to the relatively loose requirements for the content of support sessions.

These issues necessitate an effective self-management tool that can automate and standardize much of the counseling process [20]. In this regard, strategies using information and communication technology (ICT) could have great potential for the development of an effective and scalable lifestyle intervention. ICT-based interventions offer patients various benefits depending on the intervention design, including easier self-monitoring, access to health-related information, and personalized feedback, all contributing to optimizing the impact and convenience of the intervention [21,22]. They are also expected to minimize health care providers’ workload and costs [21,23-25]. Previous ICT-based interventions have been shown to successfully reduce physical parameters such as body weight (BW), BMI, and waist circumference (WC) among overweight adults [21,22,26].

### Objectives

In Japan, several companies and local governments started approaches to using ICT for the Specific Health Guidance [27,28]. However, to our knowledge, the effectiveness of ICT for the participants of the Specific Health Guidance has not been adequately evaluated in randomized controlled trials. Our primary aim is to investigate whether an app-based intervention was effective for people who are eligible for the Specific Health Guidance, the prevention program for lifestyle diseases in Japan. The authors of this study have developed an ICT-based self-management system, *DialBeticsLite*. This is a mobile app that allows the input of data on blood glucose, blood pressure (BP), BW, pedometer counts, diet, and physical exercise. It also provides users with fully automated evaluative feedback on diet modification and amount of physical exercise following the input of data each day. We provided some devices for people who use DialBeticsLite to measure their daily data. DialBetics, its former version, has been shown to successfully improve hemoglobin A_1c_ (HbA_1c_) and fasting blood glucose levels in previous trials for patients with type 2 diabetes [29,30].

## Methods

### Study Design

This study was designed to be an open-label, 2-arm, parallel-design randomized controlled trial and was conducted at the University of Tokyo Hospital, Japan. The main objective is to evaluate the efficacy of treating AO using DialBeticsLite in a population who were eligible for the Specific Health Guidance.

### Ethics Approval

The protocol and forms of informed consent were submitted to and approved by the Research Ethics Committee of The University of Tokyo Graduate School of Medicine and affiliated institutions (11696-[3]), and this study was carried out in compliance with the Declaration of Helsinki. This study was conducted among volunteer participants in a nonclinical setting, that is, a company, and was retrospectively registered at the University Hospital Medical Information Network Clinical Trials Registry (UMIN000042045).

### Participants

The selection and recruitment of participants were conducted among the employees of a securities company located in Tokyo by advertising via email and the company intranet. This company is one of the top-ranked securities companies in Japan, and the average annual salary of employees is approximately JPY ¥10,000,000 (US $85,000). We recruited participants of the Specific Health Guidance who were also smartphone users and were willing to use the ICT system. Participants of the Specific Health Guidance were aged 40 to 75 years, who also satisfied both the following conditions: (1) WC ≥85 cm (men) or WC ≥90 cm (women) or BMI ≥25 kg/m^2^ and (2) at least one of the three metabolic abnormalities from (1) hyperglycemia (fasting glucose level ≥100 mg/100 mL or HbA_1c_ ≥5.6%), (2) hypertriglyceridemia (triglycerides ≥150 mg/dL) or low high-density lipoprotein (HDL)-cholesterol (HDL <40 mg/dL), and (3) high BP (systolic BP ≥130 mm Hg or diastolicBP ≥85 mm Hg). Patients who received any medication for the treatment of hypertension, dyslipidemia, or diabetes were excluded.

All participants who met the eligibility criteria had AO (WC ≥85 cm for men or ≥90 cm for women). The WC values used had been measured for screening AO at an annual routine health checkup.

All participants provided written informed consent before the trial commenced; they were informed of their right to withdraw from the study at any time and how the data collected from the study would be used. This included an explanation of how data would be accessible to the research team to be used for analysis and dissemination, following the conclusion of the trial. Any results obtained from analyzing these data were to be presented at major domestic and international scientific conferences and submitted for peer-reviewed journals of international repute and visibility.

### Design of DialBeticsLite

**Figure 1:**
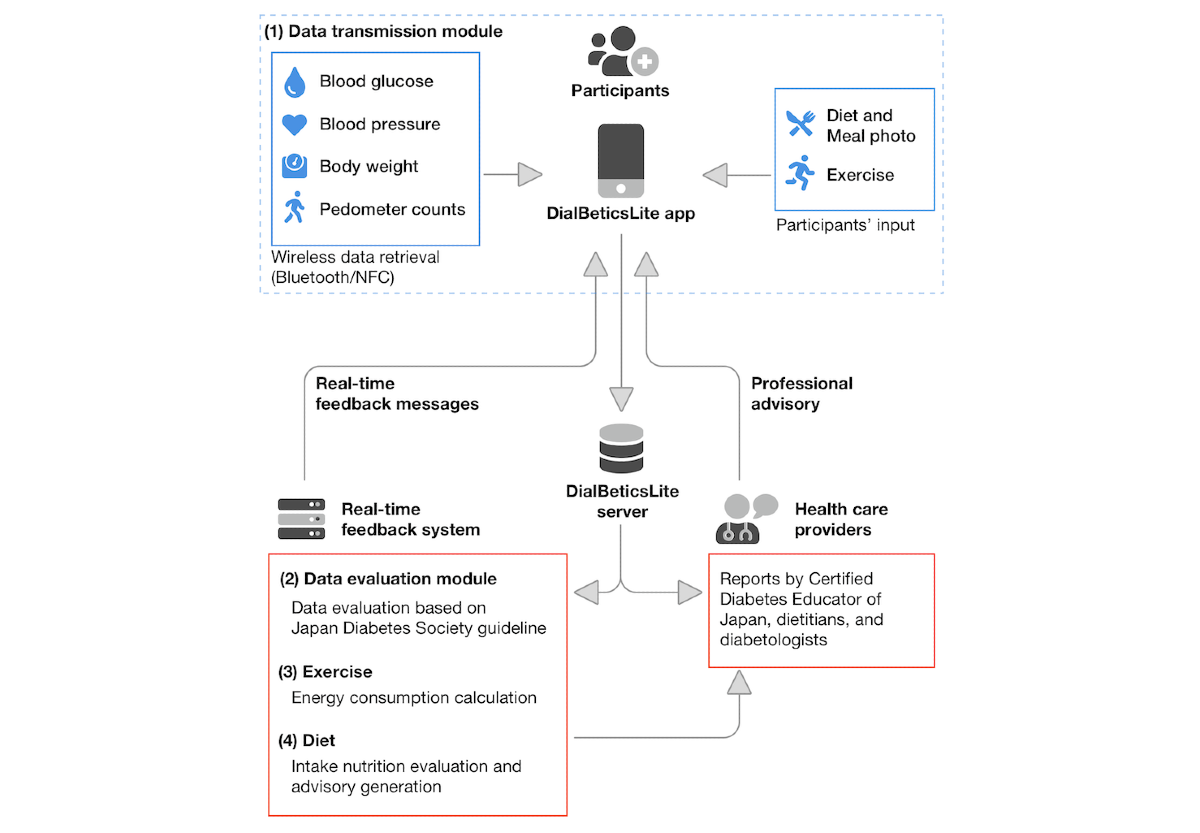
Design of DialBeticsLite. NFC: near-field communication.

The details of the DialBeticsLite system are shown in Figure 1. The app facilitated the daily recording of several physical parameters, in addition to tracking lifestyle behavior, that is, diet and exercise. Participants were asked to measure their blood glucose and BP levels twice a day at home, once after waking up in the morning and once before going to bed at night. They were also required to measure their BW in the morning. We also asked them to wear a pedometer for the entire day to measure the number of daily steps and approximate the energy expended by walking. These data were then transferred to the participant’s smartphone by near-field communication or Bluetooth and sent instantly to the server, where the data were automatically evaluated. The evaluation of pedometer counts was carried out following the Japanese official physical activity guidelines for health promotion, which sets the target count >8000 steps per day [31]. Other data including blood glucose and BP levels were evaluated based on the Japan Diabetes Society guideline’s target values [32]; desired values were set at (1) blood glucose concentrations <110 mg/dL before breakfast and <140 mg/dL at bedtime, and (2) BP with systolic BP <125 mm Hg and diastolic BP <75 mm Hg. Once DialBeticsLite determined whether each reading was satisfactory according to the guidelines, the evaluation outcomes were received by each participant’s smartphone immediately. When DialBeticsLite detected critical values including (1) blood glucose concentrations >400 mg/dL or <70 mg/dL and (2) BP with systolic BP >220 mm Hg or diastolic BP >110 mm Hg, they were automatically reported to the research team, and if deemed necessary, attending physicians were informed to contact a participant with abnormal data as appropriate.

In addition, the participants could also input dietary information (type and quantity of food, accompanied by a photograph of the meal) and details of the type and duration of physical exercise, which had been completed each day separately from pedometer counts. The app evaluated these data on lifestyle similarly to blood glucose concentrations and BP, with specific advice on diet modification sent to the participant immediately after the input of data. The app provided feedback on whether the participants’ macronutrient balance was appropriate, along with visualizations that indicated the nutritional balance of each meal. Specific guidance regarding dietary fiber and salt was also provided, in an effort to maintain intake within the recommendation set out by the Japan Diabetes Society guidelines.

### Intervention and Control

Blocked randomization within strata [33] was used to allocate participants equally to the intervention and control groups. We separated the participants into 2 BMI groups (BMI <25 kg/m^2^ and BMI ≥25 kg/m^2^), 2 visceral fat area (VFA) groups (VFA <100 cm^2^ and VFA ≥100 cm^2^), and 2 WC groups (WC <85 cm and WC ≥85 cm). This gave us 2×2×2=8 strata, and the block size in each stratum was 4. In other words, 4 participants in the same strata at a time were randomized to ensure that 2 patients were assigned to the treatment group and 2 patients were assigned to the control group.

All participants attended an educational group session at baseline, which was a 40-minute lecture at the University of Tokyo Hospital. This lecture was conducted by nurses, diabetologists, and dietitians who were all part of the research team and included information regarding diet and exercise.

The participants in the intervention group were asked to use DialBeticsLite for the next 3 months and trained on how to use the app by the research team after the lecture to ensure correct and informed use of the app (ie, how to take measurements and record them as intended, as well as how to interpret the readings). The intervention group was also provided with various other devices, including a Bluetooth-enabled BP monitor (HEM-7271T; Omron) and scale (HBF-255T; Omron), a near-filed communication–enabled glucometer (MS-FR201B; Terumo), and a pedometer (MT-KT02DZ; Terumo). These devices were all paired with a single smartphone (Galaxy Note3 SC-01F; Samsung) also provided as part of the intervention, which transmitted the readings to the DialbeticsLite server via a wireless network. For the full duration of the study, the research team provided technical assistance to the participants in the intervention group in addition to monitoring the content and frequency of daily records. The system automatically triggered an alert if participants failed to record data for >3 days, and these alerts were checked by the research team via the administrator screen. The nurse encouraged participants to restart measurements, either by email (after 1 week of inactivity) or by giving a call on the phone (after an additional week of inactivity). In cases where a participant failed to record any data (ie, not recording any of the physical parameters, food, or exercise) for 3 weeks, we deemed the participant as a dropout. As the system was solely designed for self-management and direct feedback using the input data, participants were asked to consult the physicians of their company if they had any health concerns unrelated to the intervention.

In contrast, the participants in the control group were not provided with the app or electronic devices, and they only participated in the assessment and lecture at baseline, in addition to the follow-up assessments at the end of 3 months.

### Study Outcomes and Data Collection

The primary outcome of this study was the change in VFA from baseline to the 3-month follow-up. VFA was measured by differentiating visceral fat and abdominal subcutaneous fat using the current flow from 2 routes (DUALSCAN, HDS-2000; Fukuda Colin). HDS-2000 underestimates VFA compared with computed tomography scan, but the correlation was very high (*r*=0.89) [34]. HDS-2000 can be a good alternative for evaluating VFA because of its simplicity and noninvasiveness. To prevent variation across raters in the measurement procedures, the VFA of all participants was measured by the same individual with sufficient experience. The person was unblinded for the group of intervention. Secondary outcomes were changes in both physical and metabolic parameters from baseline to the 3-month follow-up. Physical parameters included BW, WC, BMI, and BP. Metabolic parameters included cholesterol, triglyceride, fasting plasma glucose, and HbA_1c_ levels, which were measured using blood tests. The physical parameters and metabolic parameters of all participants were measured at baseline and at the 3-month follow-up at the University of Tokyo Hospital. BP was measured after the participants took two deep breaths in the sitting position, whereas WC was measured at the umbilical level in the standing position. BMI was determined by calculating the ratio of BW (kg) to height squared (m^2^). Blood tests were used to measure the concentration of fasting plasma glucose, HbA_1c_, triglyceride, total cholesterol, low-density lipoprotein (LDL) cholesterol, and HDL cholesterol.

For the intervention group, the following variables were also assessed: total energy intake (kcal), steps taken per day, the use rate of the app, and the use rates of individual functions within the app, including blood glucose, BP, BW, diet, and physical exercise.

### Statistical Analysis

The demographic characteristics and other parameters of the intervention and the control groups at baseline were presented as mean (SD) and compared using the Welch 2-tailed *t* test. To evaluate the effect of the DialbeticsLite, we used the Welch *t* test to compare the change in VFA (primary outcome) and changes in other parameters (secondary outcomes) between the 2 groups. We also conducted a linear regression analysis for both the primary and secondary outcomes, adjusted for VFA at baseline, as a post hoc test.

We also conducted several exploratory analyses to examine whether the improvement in AO was associated with lifestyle factors (pedometer counts and calorie intake) or the different use patterns of DialBeticsLite observed within the intervention group. We presented the numbers of days for which each parameter was recorded (ie, the number of days each function was used for) as the median (IQR). We calculated the Pearson correlation index and conducted tests for noncorrelation for each function to examine the relationship between the change in VFA and the use rate of each function. We plotted the trend of the average pedometer counts and calorie intake per day over the study period. We performed linear regression analysis to assess the effect of average pedometer counts and calorie intake per day on the change in VFA, adjusted for age and VFA at baseline. The retention rates of the pedometer count and diet functions were also plotted to help visualize engagement and retention over time.

A *P* value of <.05 was considered statistically significant, and all statistical analyses were performed using available-case analysis and using SAS (version 9.4; SAS Institute Inc).

## Results

### Participants

**Figure 2:**
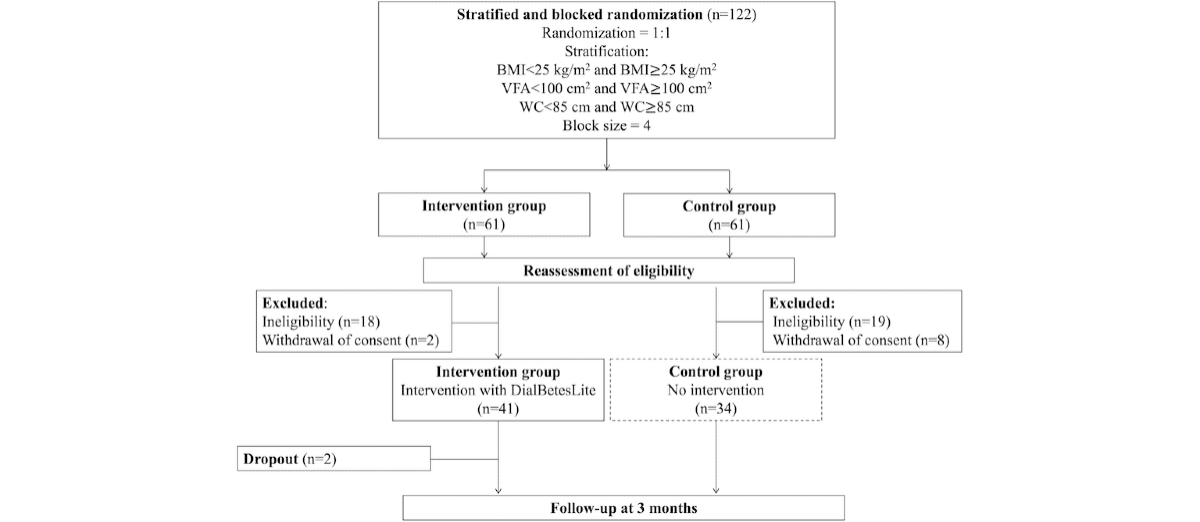
Flow diagram of the study participants. VFA: visceral fat area; WC: waist circumference.

The study was approved in September 2017, and participants were recruited. The final 3-month follow-up was completed in February 2018. As shown in Figure 2, a total of 122 participants who provided written informed consent were randomly assigned to either the intervention or control group at randomization. This initially resulted in 50% (61/122) of participants for each group. Upon reassessment of eligibility, of the 61 participants, 20 (33%) were excluded from the intervention group: 18 (30%) owing to ineligibility and 2 (3%) owing to withdrawal of consent. Similarly, for the control group, of the 61 participants, 27 (44%) were excluded: 19 (31%) owing to ineligibility and 8 (13%) owing to withdrawal of consent. Furthermore, 2 participants dropped out over the course of the trial and were excluded from the intervention group.

There was a significant difference in VFA between the intervention and control groups at baseline but no difference in the other variables (Table 1). Although there was no intention of studying well-educated men exclusively, all the participants who participated in this trial were men university graduates.

**Table 1:** Baseline characteristics of participants.

Variable		Intervention group (n=41)	Control group (n=34)	*P* value
Age (years), mean (SD)		49.3 (6.1)	48.5 (5.3)	.55
**Sex, n (%)**				
	Men	41 (100)	34 (100)	—^a^
	Women	0 (0)	0 (0)	—
**Physical parameters, mean (SD)**				
	Visceral fat area (cm^2^)	118.9 (32.3)	105.4 (20.8)	.03
	Height (cm)	172.2 (5.0)	171.3 (6.5)	.49
	Body weight (kg)	81.3 (9.9)	78.0 (6.8)	.09
	BMI (kg/m^2^)	27.4 (3.0)	26.6 (2.2)	.19
	Waist circumference (cm)	96.1 (7.7)	95.0 (9.2)	.57
**BP^b^, mean (SD)**				
	Systolic BP (mm Hg)	130.7 (10.7)	128.2 (10.2)	.32
	Diastolic BP (mm Hg)	86.3 (8.4)	86.9 (7.3)	.76
**Lipid metabolism and glucose, mean (SD)**				
	Total cholesterol (mg/dL)	217.8 (30.4)	221.0 (37.3)	.69
	LDL^c^ cholesterol (mg/dL)	137.7 (24.0)	141.1 (34.8)	.64
	HDL^d^ cholesterol (mg/dL)	51.5 (10.0)	52.2 (11.2)	.76
	Triglyceride (mg/dL)	142.9 (75.9)	138.3 (78.7)	.80
	Fasting plasma glucose (mg/dL)	88.9 (12.1)	89.5 (7.7)	.80
	HbA_1c_^e^ (%)	5.5 (0.3)	5.5 (0.3)	.85

^a^Comparisons of sex were not conducted because all participants were males.

^b^BP: blood pressure.

^c^LDL: low-density lipoprotein.

^d^HDL: high-density lipoprotein.

^e^HbA_1c_: hemoglobin A_1c_.

### Changes in VFA and Physical and Metabolic Parameters

At the 3-month follow-up, although the control group observed an increase in VFA (mean +1.9, SD 16.2 cm^2^), participants in the intervention group lost considerable amounts of VFA (mean −23.5, SD 20.6 cm^2^). Consequently, those who underwent the intervention had statistically significant reductions in the primary outcome compared with those in the control group (*P*<.001). This finding did not change after adjusting for VFA at baseline between the 2 groups as a post hoc test (Table 2).

In addition, the intervention group displayed a statistically significant improvement in BW (mean −3.0, SD 2.8 kg vs mean+1.1, SD 1.6 kg; *P*<.001), BMI (mean −1.0, SD 1.0 kg/m^2^ vs mean+0.4, SD 1.6 kg/m^2^; *P*<.001), and WC (mean −4.8, SD 3.8 cm vs mean −1.6, SD 7.7 cm; *P*=.04) compared with those in the control group. For all of the remaining physical and metabolic parameters that were assessed, including BP, cholesterol, triglycerides, fasting plasma glucose, and HbA_1c_, no differences between the 2 groups were observed (Table 2).

Further analysis revealed that the reduction in VFA in the intervention group was significantly associated with both the number of steps per day (*P*<.001) and calorie intake per day (*P*<.001), after results were adjusted to take age and the VFA at baseline into consideration (Table 3).

**Table 2:** Change in parameters.

Variable		Intervention group (n=39), mean (SD)	Control group (n=34), mean (SD)	*P* value	Adjusted *P* value^a^
**Physical parameters**					
	Visceral fat area (cm^2^)	−23.5 (20.6)	1.9 (16.2)	<.001	<.001
	Body weight (kg)	−3.0 (2.8)	1.1 (1.6)	<.001	<.001
	BMI (kg/m^2^)	−1.0 (1.0)	0.4 (0.6)	<.001	<.001
	Waist circumference (cm)	−4.8 (3.8)	−1.6 (7.7)	.04	.02
**BP^b^**					
	Systolic BP (mm Hg)	−3.9 (10.1)	−1.4 (9.6)	.29	.28
	Diastolic BP (mm Hg)	0.1 (9.0)	0.9 (8.5)	.68	.61
**Lipid metabolism and glucose**					
	Total cholesterol (mg/dL)	−4.1 (20.1)	2.0 (21.7)	.22	.33
	LDL^c^ cholesterol (mg/dL)	−3.3 (16.1)	−1.6 (21.6)	.72	.53
	HDL^d^ cholesterol (mg/dL)	4.5 (7.9)	1.4 (5.7)	.06	.07
	Triglyceride (mg/dL)	−26.4 (50.5)	11.5 (88.3)	.03	.11
	Fasting plasma glucose^e^ (mg/dL)	1.1 (6.7)	2.7 (7.3)	.35	.55
	HbA_1c_^f^ (%)	0.0 (0.1)	0.0 (0.1)	.72	.97

^a^Adjusted for visceral fat area at baseline.

^b^BP: blood pressure.

^c^LDL: low-density lipoprotein.

^d^HDL: high-density lipoprotein.

^e^Because 1 participant was absent in the control group, the number of participants analyzed was 39 in the intervention group and 33 in the control group.

^f^HbA_1c_: hemoglobin A_1c_.

**Table 3:** Linear regression for the change in visceral fat area in the intervention group.

Variable	Estimate	95% CI	*P* value
Number of steps per day (every 1000 steps)	−4.76	−6.59 to −2.92	<.001
Calorie intake per day (every 100 kcal)	2.29	1.00 to 3.58	<.001
Age (years)	−0.33	−1.14 to 0.48	.42
Visceral fat area at baseline (cm^2^)	−0.11	−0.25 to 0.03	.13

### Changes of Number of Steps and Calorie Intake During the Study Period

**Figure 3:**
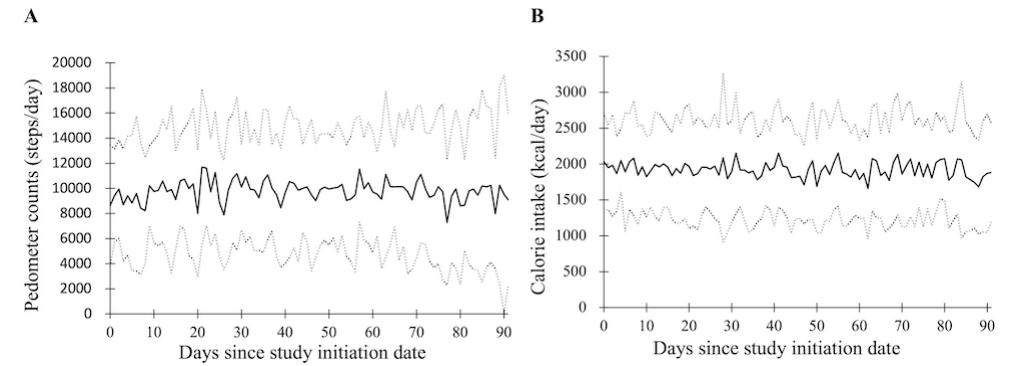
Variables of lifestyle behavior recorded during the study period. (A) Number of steps. Solid line: daily mean pedometer counts; dotted lines: mean (SD). (B) Calorie intake. Solid line: daily mean calorie intake; dotted lines: mean (SD).

In the intervention group, participants walked an average of nearly 8000 (SD 5139) steps (Figure 3). Similarly, the average calorie intake was nearly 2000 (SD 703) kcal/day (Figure 3). Both parameters remained almost constant during the study period.

### Use of DialBeticsLite in the Intervention Group

**Figure 4:**
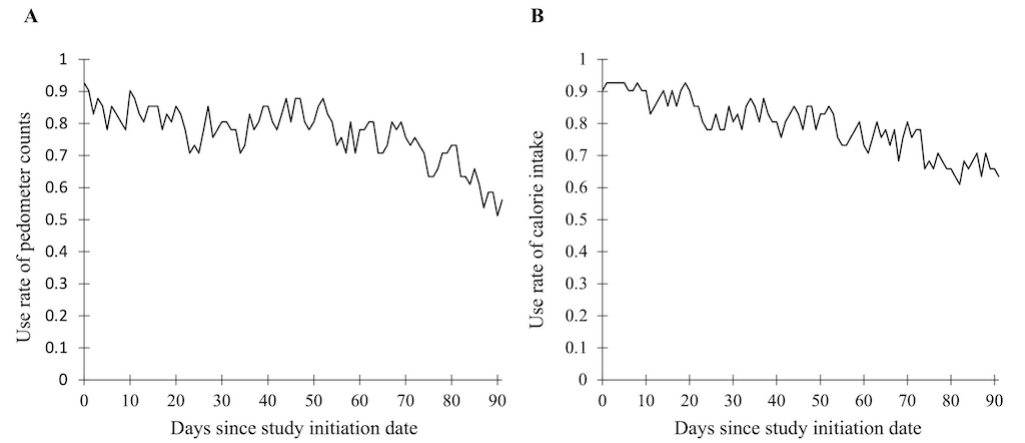
Retention rates of the functions regarding lifestyle behavior during study period. (A) Pedometer counts and (B) calorie intake.

By analyzing the data recorded by DialBeticsLite, we found the median use rate of the app to be 100% (IQR 98%-100%) within the intervention group (Table 4). When examining individual functions, differences in use rates were observed. The use rates of the self-monitoring functions for BW (median 93%, IQR 73%-98%), pedometer counts (median 85%, IQR 74%-93%), BP before breakfast (median 88%, IQR 61%-95%), breakfast (median 98%, IQR 78%-100%), lunch (median 97%, IQR 62%-99%), and dinner (median 95%, IQR 75%-99%) were high, whereas those regarding exercise input (median 1%, IQR 0%-14%), BP at bedtime (median 61%, IQR 29%-86%), blood glucose (median 0%, IQR 0%-0%), and snacks (median 20%, IQR 10%-36%) were low. We examined the use of functions that recorded lifestyle and behavior, specifically the pedometer count function and the diet function (including breakfast, lunch, dinner, and snacks). We found that both the retention rate of the pedometer counts function and diet function decreased over time during the study, with the former declining from 90% to 50% and the latter declining from 90% to 70% (Figure 4). We analyzed the recorded data by producing a Pearson correlation matrix to determine whether any of the individual function’s use rates showed a correlation with the reduction of VFA (Multimedia Appendix 1). We found that the BW (*r*=−0.27, *P*=.10), pedometer counts (*r*=−0.25, *P*=.13), BP before breakfast (*r*=−0.27, *P*=.10), and snacks (*r*=−0.27, *P*=.10) functions showed a positive correlation with the reduction of VFA, although this was not statistically significant. In contrast, a significant correlation was observed between the use rates of the functions. Correlation coefficients of over 0.9 were obtained between the BW and BP before breakfast functions, as well as between the individual diet functions, including breakfast and lunch, breakfast and dinner, and lunch and dinner.

**Table 4:** Number of days with recordings and use rate for each parameter in the intervention group.

Variable		Days with recordings (n=92), median (IQR)	Use rate (%), median (IQR)
Total^a^		92 (90-92)	100 (98-100)
Body weight		86 (67-90)	93 (73-98)
Pedometer counts		78 (68-86)	85 (74-93)
Exercise input		1 (0-13)	1 (0-14)
**Blood pressure**			
	Before breakfast	81 (56-87)	88 (61-95)
	At bedtime	56 (27-79)	61 (29-86)
**Blood glucose**			
	Before breakfast	0 (0-0)	0 (0-0)
	At bedtime	0 (0-0)	0 (0-0)
**Calorie intake**			
	Breakfast	90 (72-92)	98 (78-100)
	Lunch	89 (57-91)	97 (62-99)
	Dinner	87 (69-91)	95 (75-99)
	Snacks	18 (9-33)	20 (10-36)

^a^The median number of days with at least one variable recorded.

## Discussion

### Principal Findings

In this randomized clinical trial, we examined the effect of using DialBeticsLite for approximately 3 months in addition to traditional counseling for the participants of the Specific Health Guidance with AO. Statistically significant reductions were observed in VFA, which was the primary end point, and some other parameters (BW, BMI, and WC). These results suggest that the addition of mHealth interventions could make the Specific Health Guidance more beneficial. According to an exploratory analysis within the intervention group, the mean number of steps and calorie intake per day during the study period were associated with a decrease in VFA.

We observed a significant reduction for VFA, BW, BMI, and WC after the intervention, which is consistent with other studies that have shown that mHealth interventions can reduce BW, BMI, and WC [21,22,26]. DialBeticsLite encourages self-monitoring through daily records and provides personalized advice. Frequent self-weighing and recording of daily weight patterns by patients with obesity has been reported to be effective in weight loss programs [35]. Our findings also reinforce the effectiveness of self-monitoring for individuals with AO. Owing to the nature of the intervention, its impact relies on engagement [36]. Previous studies have indicated so, reporting a significant association between weight loss and the use rate of self-monitoring functions or the use rate of an app in general [36,37]. The same was observed for behavior, with participants with higher app use exhibiting increased physical activity and decreased caloric intake [37]. In this study, although strong correlations were not observed between the loss of VFA and the use of self-monitoring functions, they do not necessarily contradict previous studies. Engagement in the intervention group was higher than that in other studies involving behavioral self-monitoring with mHealth, as the median number of days of full app use was 92 out of 92 days [22,38]. Similarly, a median of over 80 days for both BW and diet self-monitoring functions (excluding snacks) was observed, which was also higher than that in past trials [29]. The higher engagements may have resulted in difference of reduction of VFA between the intervention and control groups.

Another important finding, but perhaps least surprising, was that VFA reduction was associated with the number of steps taken and calorie intake in the intervention group. Increasing the number of steps and decreasing the number of calories are natural ways to improve AO. In the intervention group, participants walked an average of nearly 8000 steps (Figure 3), which seems to be sufficient, but an increase in the number of steps may still be effective. Similarly, further reduction of calorie intake from 2000 kcal/day (Figure 3) may further improve AO. Although some randomized controlled trials have shown that mHealth interventions are effective [21,22,26], few studies have revealed the cause of the improvement. Our findings may support the idea that we should focus not only on increasing engagement but also on causing behavior change.

In contrast to the significant reduction for VFA, BW, BMI, and WC after the intervention, all remaining physical and metabolic parameters, including BP, cholesterol, triglycerides, fasting plasma glucose, and HbA_1c_, showed no differences between the 2 groups. However, because these parameters were well controlled and almost within the normal range at baseline, improvement may have been difficult regardless of the intervention.

Although mHealth allows for easier initiation and recruitment, it is also more prone to disengagement [39]. Over the course of this study, the retention rates declined within the intervention group (Figure 4). This is also seen in other studies, and there is a need to explore potential methods to increase engagement and retention to maximize the benefits and efficacy of mHealth as a replacement for in-person support [36]. A study examining the reasons for dropout identified that daily recording could become overly repetitive and burdensome, with too much data to track [38], whereas another study targeting patients who had been prescribed medication discovered that the time and effort required were the predominant barriers to using personal health record systems [40]. In contrast, the frequent use of self-monitoring functions has been shown to increase retention [41], and low engagement has been identified as a predictor for participants abandoning the app [42]. Therefore, retention and adherence could potentially be improved by identifying specific functions of the app with higher use rates and associations with positive outcomes while eliminating other features to simplify the app. For instance, in our study, the recording function for blood glucose was rarely used and may have been useless. In addition, although not statistically significant, the BW, pedometer counts, BP before breakfast, and snacks functions showed a positive correlation with the reduction of VFA. The high measurement rates of this study population might have resulted in an insignificant correlation; placing further emphasis on and developing these functions might further improve the effect of the intervention on AO. Furthermore, the use rates of some functions were strongly associated with those of other functions. For example, those who used the BW function were more likely to have been using the BP before breakfast function, and the same was seen between the individual diet functions, including breakfast and lunch, breakfast and dinner, and lunch and dinner. To optimize the app design, further detailed investigations into the effect of each feature are required.

### Limitations

Although the results of this study are promising, they should be interpreted with some caution, as there are several limitations to this study. First, past studies have revealed that for studies examining the effect of lifestyle interventions, those with sample sizes of >90 and intervention durations of >8 weeks tend to have more successful outcomes [36]. Although, at randomization, our study included 122 individuals, only 73 (59.8%) were included in the final analysis of results, and this relatively small sample included only Japanese men aged 40 to 75 years. Many patients were found to not meet the eligibility criteria after allocation because of our lack of confirmation; assessment was determined only by the information obtained before allocation. Thus, the transportability of the results to other populations, including those with women and different ethnic groups, is limited. As there has been evidence from a recent study to support a difference in retention and willingness to use mHealth interventions between men and women, with the latter being more reluctant to use apps intended to treat diabetes [43], there is a need to assess whether DialBeticsLite also has an uneven adherence between the 2 sexes.

Second, the participants were observed for a relatively short period of 3 months, and although this is longer than 8 weeks, the long-term impact of the intervention remains to be tested. This is especially important considering that many participants in lifestyle interventions struggle to maintain the significant physical and behavior changes achieved at the end of the intervention [44,45]. There has been evidence of the effect of mHealth being relatively short-lived, with participants regaining any weight they had lost during the intervention period [46].

Third, despite using stratified randomization, VFA differed between the 2 groups at baseline. As we categorized the VFA and allocated participants by stratified randomization, this phenomenon has some probability of occurrence. However, a sensitivity analysis to examine the influence of this difference was conducted during the analysis of primary and secondary outcomes, and as the results were relatively unchanged, we concluded that the difference in VFA at baseline had little influence on the primary and secondary outcomes.

Fourth, the provision of some medical devices to the intervention group may have influenced the results obtained by improving the participants’ motivation. Therefore, it may be more appropriate to interpret the results of this study as indicative of the combined effect of the app and the distribution of medical devices, rather than the effect of the app alone.

Finally, as daily steps and caloric intake were not recorded in the control group, we cannot be certain of the extent to which these 2 factors were affected by the intervention. Although physical parameters improved significantly, we were unable to observe a significant reduction in total energy intake or an increase in physical activity during the intervention. At first glance, this may suggest that the intervention did not contribute to improving lifestyle and behavior; however, considering a previous study that showed that mHealth intervention prevented the reduction of daily steps, this may not be the case [47]. Although this previous study did not produce statistically significant results, likely owing to its small sample size, it suggests that an increase in daily steps is not necessary for an mHealth intervention to be meaningful. Allowing participants to maintain a high level of physical exercise in itself is an achievement and may explain why our study saw a significant decrease in BW, BMI, and VFA despite seeing no significant change for daily steps and total caloric intake.

### Conclusions

Our study suggested that DialBeticsLite, a mobile app designed to assist self-management, was feasible among Japanese adults who were eligible for the Specific Health Guidance. The mHealth intervention resulted in a statistically significant reduction in VFA, BW, BMI, and WC over the course of 3 months. Compared with traditional methods, ICT systems offer greater scalability and convenience and could be more cost-effective. Thus, these findings are promising and show that mHealth interventions have great potential for treating patients with AO.

## Appendix

Multimedia Appendix 1 Pearson correlation matrix for the change in visceral fat area and number of days with recordings for each parameter in the intervention group.

Multimedia Appendix 2 CONSORT-eHEALTH checklist (V1.6.1).

## Abbreviations

AO: abdominal obesity
BP: blood pressure
BW: body weight
HbA_1c_: hemoglobin A_1c_
ICT: information and communication technology
mHealth: mobile health
VFA: visceral fat area
WC: waist circumference
HDL: high-density lipoprotein
LDL: low-density lipoprotein
