# Supplementary material for: Effect of Information and Communication Technology–Based Self-management System DialBeticsLite on Treating Abdominal Obesity in the Specific Health Guidance in Japan: Randomized Controlled Trial
Source: JMIR Form Res. 2022 Mar 24;6(3):e33852. doi: 10.2196/33852 (PMC8990341; doi:10.2196/33852)
Supplement: Multimedia Appendix 1 [file formative_v6i3e33852_app1.pdf]

Multimedia Appendix1 . Pearson correlation matrix for the change in VFA and number of days with recordings for each parameter in intervention group

						**BP**		**BG**		**Food**			
Variable	Change in VFA	Total	Body weight	Pedometer counts	Exercise	Before Breakfast	At bedtime	Before Breakfast	At bedtime	Breakfast	Lunch	Dinner	Snack
Change in VFA	1	-0.03	-0.27	-0.25	-0.04	-0.27	-0.17	0.16	0.22	-0.02	-0.03	-0.10	-027
Total [a]		1	0.74**	0.73**	0.15	0.61**	0.44**	0.08	0.01	0.81**	0.76**	0.81**	0.26
Body weight			1	0.82**	0.28	0.91**	0.63**	-0.16	-0.26	0.78**	0.86**	0.81**	0.41**
Pedometer				1	0.23	0.77**	0.61**	-0.15	-0.19	0.65**	0.73**	0.70**	0.32*
Exercise					1	0.33*	0.21	-0.03	-0.11	0.26	0.28	0.25	0.19
BP: Before Breakfast						1	0.63**	-0.09	-0.22	0.70**	0.76**	0.73**	0.45**
BP: At bedtime							1	-0.31	-0.20	0.59**	0.64**	0.62**	0.35*
BG: Before Breakfast								1	0.45**	0.06	-0.11	-0.05	-0.12
BG: At bedtime									1	-0.11	-0.18	-0.17	-0.04
Food: Breakfast										1	0.96**	0.97**	0.37*
Food: Lunch											1	0.95**	0.41**
Food: Dinner												1	0.39*
Food: Snack													1

VFA, Visceral fat area; BP, blood pressure; BG, blood glucose.

[a] The number of days with at least one variable recorded.

** *P*<.01, * *P*<.05.
